# Validation of the cognitive fusion questionnaire in regular meditators and persons with schizophrenia spectrum disorders

**DOI:** 10.3389/fpsyg.2026.1748422

**Published:** 2026-04-02

**Authors:** Niklas Bergmann, Ingmar Conell, Inge Hahne, Marco Zierhut, Thi Minh Tam Ta, Eric Hahn, Kerem Böge

**Affiliations:** 1 Department of Psychiatry and Psychotherapy, Campus Benjamin Franklin, Charité – Universitätsmedizin Berlin, Corporate Member of Freie Universität Berlin, Humboldt-Universität zu Berlin, and Berlin Institute of Health, Berlin, Germany; 2 BIH Charité Junior Clinician Scientist Program, BIH Biomedical Innovation Academy, Berlin Institute of Health at Charité – Universitätsmedizin Berlin, Berlin, Germany; 3 German Center of Mental Health (DZPG), Berlin/Potsdam, Germany; 4 Department of Psychology, Medical University Brandenburg, Neuruppin, Germany

**Keywords:** cognitive fusion, meditation, psychological flexibility, schizophrenia spectrum disorders, validation

## Abstract

Cognitive fusion, a key construct in Acceptance and Commitment Therapy (ACT), describes the overidentification with internal thoughts that compromises psychological flexibility and contributes to maladaptive emotion regulation. Cognitive defusion techniques aim to reduce this entanglement, promoting adaptive functioning and improved mental health. This study examined the psychometric properties of the Cognitive Fusion Questionnaire (CFQ) across diverse populations, focusing on reliability, validity, and sensitivity to change. Data were drawn from three studies involving individuals with schizophrenia spectrum disorders (SSD) and non-clinical meditators. In Study 1, regular meditators (*N* = 779) completed the original English CFQ. Study 2 assessed the German version (CFQ-D) in patients with SSD (*N* = 123). Study 3, a randomized controlled trial (*N* = 38), evaluated the CFQ-D's sensitivity to mindfulness-based group therapy (MBGT). Internal consistency, correlations with the Southampton Mindfulness Questionnaire (SMQ), and pre–post changes were analyzed. The English CFQ showed excellent internal consistency (α = 0.94) and strong negative correlations with the SMQ (*r* = −0.67). The CFQ-D demonstrated high reliability (α = 0.92) and moderate negative correlations with SMQ (*r* = −0.30). In Study 3, participants receiving mindfulness-based therapy showed reduced cognitive fusion over time. The CFQ is a reliable, valid measure of cognitive fusion across clinical and non-clinical samples.

## 1 Introduction

The emergence of the third wave of cognitive behavioral therapies, including mindfulness- and acceptance-based approaches (Hayes et al., 1999; Kabat-Zinn, 1990; Shapiro et al., 2008), has gained considerable traction in both clinical research and therapeutic practice (Hayes and Hofmann, 2017). Acceptance and Commitment Therapy (ACT; Hayes et al., 2012) assumes that an individual's reluctance to face unpleasant psychological experiences affects their ability to cope with and respond to everyday challenges. Avoidance of negatively connoted internal events prompts individuals to engage in behaviors aimed at immediate relief, often at the expense of long-term well-being. Cognitive defusion refers to processes that reduce the literal influence of verbal events on behavior. Rather than targeting internal experiences themselves, defusion techniques aim to alter the *functional relationship* between language and action by increasing psychological distance from thoughts, attitudes, rules, and evaluative language. This shift allows individuals to respond more flexibly to situational demands rather than behaving under the automatic control of verbal processes (Hayes et al., 2006a,b). Among the six main mechanisms of ACT is cognitive defusion (Hayes et al., 2006a), which reduces the tendency for behavior to be overly influenced by internal experiences. Cognitive fusion describes a process in which thoughts exert control over behavior. While fusion can be adaptive when verbal rules support effective action, it becomes maladaptive when rigid adherence to thoughts reduces psychological flexibility and has been linked to poorer mental health outcomes, such as depression and anxiety (Lucena Santos et al., 2017; Viskovich and Pakenham, 2020). Thus, fusion is best understood in terms of workability: whether following a thought moves a person toward or away from valued outcomes. Furthermore, cognitive fusion is related to negative affect, as shown by the effects of ACT treatment (Hayes et al., 2011) and is characterized by rigidity in affective disorders and overall maladaptive emotion regulation strategies (Gabrys et al., 2018; Genet et al., 2013; Gillanders et al., 2014). Addressing cognitive fusion through therapeutic interventions like ACT and mindfulness-based approaches can enhance psychological flexibility, which is crucial for improving mental health outcomes (Hayes et al., 2012).

The Cognitive Fusion Questionnaire (CFQ), developed by Gillanders et al. (2014), was designed to measure cognitive fusion as a process. It is brief, not specific to a particular population or content, and can be used without knowledge of the construct. The CFQ exhibited a one-factor structure with excellent model fit in different samples and showed high reliability, validity, and sensitivity to treatment (Gillanders et al., 2014). Additionally, the CFQ has been applied in clinical populations, including individuals experiencing depression, anxiety, and other psychological disorders (McCracken et al., 2014). In addition to the original English version, validated translations of the CFQ are available in Brazilian-Portuguese (Lucena Santos et al., 2017), Spanish (Romero Moreno et al., 2014), Catalan (Solé et al., 2016), Korean (Kim and Cho, 2015) and French (Dionne et al., 2016). A German version was validated in a chronic pain and non-clinical sample and showed good psychometric properties (China et al., 2018). While the validity in a chronic pain population appears solid, the CFQ has not yet been validated for use in individuals with schizophrenia spectrum disorders (SSD). Therefore, further studies are needed to transfer the results to other populations.

Regular meditation has been associated with reduced cognitive fusion and increased psychological flexibility (Shapiro et al., 2008). Evaluating the CFQ in a meditator sample will provide insights into its applicability and reliability among individuals who engage in regular mindfulness practices.

The present study aimed to assess the psychometric properties of the English version of the CFQ in a large international, non-clinical sample of regular meditators. Moreover, it seeks to evaluate the psychometric properties of the German CFQ in a clinical sample of persons with schizophrenia spectrum disorders as well as convergent validity in both samples. It was hypothesized that the unidimensional structure of the original validation is confirmed (Gillanders et al., 2014). Furthermore, a significant correlation with Southampton Mindfulness Questionnaire (SMQ) (Böge et al., 2021; Chadwick et al., 2008) is hypothesized. At last, it is hypothesized that the CFQ detects changes after participation in a mindfulness-based intervention. The use of three samples and two language versions allows for a comprehensive evaluation of the CFQ across distinct contexts. The meditator sample provides a large, non-clinical benchmark for the English version. The SSD samples allow examination of the German CFQ in a clinical population characterized by cognitive and metacognitive challenges. Together, these samples enable a coherent assessment of reliability, validity, and sensitivity to change across populations that differ in psychological flexibility and linguistic processing.

## 2 Materials and methods

To test the reliability, factor structure and convergent validity of the original CFQ, a large international, non-clinical sample of regular meditators was recruited via online recruitment (Study 1). In addition, reliability, factor structure and convergent validity of the German CFQ (CFQ-D) were examined in a sample of persons with schizophrenia spectrum disorders (Study 2) and longitudinal data from a clinical intervention study (Study 3) to determine treatment sensitivity. All studies were approved by the ethics committee of Charité - Universitätsmedizin Berlin.

### 2.1 Study 1: reliability, factor structure, and convergent validity of the CFQ in an international, non-clinical sample of meditators

#### 2.1.1 Participants and procedure

Recruitment was conducted via online platforms, using an online questionnaire on Unipark Software's Questback survey platform, dedicated to meditation, including directories of meditation teachers and various meditation communities and organizations. A brief study description and an invitation to participate were posted on these platforms. Participants completed a variety of questionnaires assessing prevalence, predictors and types of unpleasant and adverse effects of meditation in regular meditators in approximately 20–25 min. For a detailed overview of measures please refer to Pauly et al. (2021).

The study involved *N* = 779 adult participants recruited between June 2020 and August 2021 from a group of meditators. In this study, “meditators” were defined as individuals with at least 1 month of regular meditation experience. This cutoff has been used in previous research to ensure a basic level of familiarity with mindfulness practices while still allowing for variability in experience (Schlosser et al., 2019; Pux et al., 2022). Using this criterion helps capture a group with sufficient exposure to heterogeneous contemplative techniques to meaningfully engage with constructs such as cognitive fusion and mindfulness, without restricting the sample to highly experienced practitioners. Of the 950 respondents, 171 were excluded due to insufficient meditation experience of less than a month (*n* = 155), being younger than 18 years (*n* = 11) or claiming to meditate for an unrealistic amount of month (*n* = 5).

Participants in the meditator sample were on average 39.8 years old (*SD* = 16.4) and predominantly female. Most resided in Europe or North America, with additional representation from other regions of the world. Meditation experience varied, with participants reporting an average of 4.8 weekly meditation hours and 76.7 months of lifetime practice. Detailed demographic information is presented in Table 1.

**Table 1:** Sociodemographic information of study 1.

Measure	Value
Age (years), *M* (*SD*)	39.8 (16.4)
Gender	
Female	69.8%
Male	28.7%
Diverse	1.5%
Region of residency	
Europe	52.5%
North America	30.6%
Australia and New Zealand	8.5%
Asia	6.9%
South America	0.9%
Africa	0.6%
Meditation experience	
Weekly meditation hours, *M* (*SD*)	4.8 (13.2)
Meditation experience (months), *M* (*SD*)	76.7 (118.1)

#### 2.1.2 Measurements

Cognitive Fusion. The original Cognitive Fusion Questionnaire (CFQ; Gillanders et al., 2014) was used for the study. This self-report instrument consists of seven items, each rated on a 7-point Likert scale from “always true” (7) to “never true” (1). The total score ranges from 7 to 49, with higher scores reflecting greater cognitive fusion, which means less psychological flexibility. Previous research has shown that the CFQ has excellent internal consistency with a Cronbach's alpha (α) = 0.95 (Gillanders et al., 2014).

Mindfulness. The Southampton Mindfulness Questionnaire (SMQ) was used to assess mindfulness. The SMQ consists of 16 items rated on a six-point Likert scale from “strongly agree” (6) to “strongly disagree” (0), giving a total range of 0 to 96. The SMQ is conceptualized in four interrelated constructs that include (1) decentered awareness, (2) letting go, (3) non-judgment, and (4) non-aversion. Half of the items are positively worded, the other half negatively. It has demonstrated robust psychometric properties, with an internal consistency of α = 0.89 (Chadwick et al., 2008). In this study, the SMQ displayed an α = 0.92.

#### 2.1.3 Statistical analysis

All analyses were conducted with IBM SPSS Statistics version 30 and R version 4.2.2 using the package *lavaan* (Rosseel, 2012). Pearson's correlation coefficient (*r*) was calculated for the CFQ and SMQ scores to determine convergent validity. Model fit indices considered included chi-square (χ^2^), root mean square error of approximation (RMSEA), confirmatory factor index (CFI), and standardized root mean square residual (SRMR; Kline, 2015). The sample size was chosen to ensure stable factor analytic results, as guidelines suggest that samples above 500 are considered “very good” to “excellent” for structural validation (Comrey and Lee, 1992; Kline, 2015). All analyses were performed with a significance level of α = 0.05.

### 2.2 Study 2: reliability, factor structure and convergent validity of the CFQ in persons with schizophrenia spectrum disorders

#### 2.2.1 Participants and procedure

Participants were recruited via the inpatient ward for psychotic disorders and the outpatient facility of the Department of Psychiatry and Neurosciences at Charité – Universitätsmedizin Berlin, Campus Benjamin Franklin. Therefore, the sample included participants under acute treatment as well as patients under long-term treatment. Eligible participants were 18–65 years of age, diagnosed with an F2x.x spectrum diagnosis according to ICD-10 criteria by a licensed psychiatrist, and provided informed consent. Participants were excluded for severe psychotic symptoms, based on a score of seven on the positive scale of the Positive and Negative Syndrome Scale (PANSS) assessed prior to recruitment. Other exclusion criteria comprised neurological disorders or acute substance misuse besides nicotine, assessed by a licensed psychiatrist. The mean age of the sample was 42 years (*SD* = 14). Most participants identified as male, with smaller numbers identifying as female or diverse. The majority held German nationality, while a smaller subset reported Turkish, Vietnamese, or other nationalities. In terms of diagnostic status, most individuals were diagnosed with schizophrenia (F20), followed by schizoaffective disorder (F25) and a small number with other psychotic disorders. Detailed demographic information is presented in Table 2.

**Table 2:** Sociodemographic information of study 2.

Measure	Value
Age (years), *M* (*SD*)	42 (14)
Gender	
Male	74
Female	47
Diverse	1
Unknown	1
Nationality	
German	104
Turkish	4
Vietnamese	5
Other	10
Marital status	
Unmarried	91
Married	11
Divorced	20
Widowed	1
Diagnosis (ICD-10)	
F20—schizophrenia	94
F21—schizotypal disorder	2
F22—persistent delusional disorder	1
F23—acute/transient psychotic disorder	5
F25—schizoaffective disorder	20
F29—unspecified psychosis	1

For Study 2, the CFQ and SMQ were administered as part of a broader assessment battery used across several other measurement validation or clinical studies (Böge et al., 2021, 2024; Hahne et al., 2026, 2025). Participants completed a set of self-report questionnaires, including demographic items, within a single assessment session. The full battery varied slightly across studies, but the CFQ and SMQ were consistently included to allow a validation.

#### 2.2.2 Measures and statistical analysis

The German versions of the previously described questionnaires have been employed (SMQ-D: Böge et al., 2021; CFQ-D: China et al., 2018). For the SMQ, an α = 0.70 was found. The same analyses as in Study 1 have been conducted. Recruitment targeted a minimum of 100 participants, which is considered adequate for reliability and validity testing in clinical populations (DeVellis, 2016). The achieved sample of *N* = 123 aligns with comparable validation studies in psychiatric samples (Vilardaga et al., 2009).

### 2.3 Study 3: treatment sensitivity in a schizophrenia spectrum disorder sample

#### 2.3.1 Participants and procedure

A total of *N* = 40 inpatients with schizophrenia spectrum disorders were included in the study. Participants were recruited from the inpatient ward and the psychiatric day clinic at the Department of Psychiatry and Psychotherapy, Charité - Universitätsmedizin Berlin, Campus Benjamin Franklin. Eligibility criteria were similar to study 2. In Study 3, the CFQ was administered as part of a broader assessment battery within a feasibility study evaluating mindfulness-based group therapy (Böge et al., 2021). Participants completed several self-report questionnaires, including demographic items, during a single assessment session. The CFQ was included specifically for the purpose of the present validation. Patients were recruited from an inpatient psychiatric service providing acute and subacute treatment for schizophrenia spectrum disorders. Participants were randomly assigned to receive either mindfulness-based group therapy (MBGT) in addition to the treatment as usual (MBGT + TAU) (*n* = 21) or TAU alone (*n* = 19). Assessments were conducted at baseline (T0) and post-intervention (T1, after 4 weeks). Two participants did not complete the post-intervention assessment, resulting in *n* = 20 participants completing both assessment points in MBGT + TAU and *n* = 18 in the TAU group.

The mean age of the sample was 40.1 years (*SD* = 13.6). The majority of participants were male (*n* = 24), with the remainder identifying as female (*n* = 16). Most individuals were German nationals, with a small number reporting another nationality. Regarding diagnostic status, most participants met criteria for schizophrenia (F20), while a smaller subset was diagnosed with schizoaffective disorder (F25). Detailed demographic information is presented in Table 3.

**Table 3:** Sociodemographic information of study 3.

Measure	MBGT + TAU	TAU
Age (years), *M* (*SD*)	37.71 (12.82)	42.74 (14.11)
Gender		
Male	11	13
Female	10	6
Nationality		
German	17	18
Other	4	1
Primary diagnosis		
F20 (schizophrenia)	20	18
F25 (schizoaffective disorder)	1	1

#### 2.3.2 Intervention

MBGT took place over 4 weeks, with three therapy sessions per week. The sessions focused on core aspects of mindfulness, such as breath awareness, sensory awareness, detachment, and body awareness. A psychotherapist with extensive experience in mindfulness-based practices led the sessions, supported by a psychologist trained in cognitive behavioral therapy (Böge et al., 2021; Böge and Hahn, 2021).

#### 2.3.3 Measures

The primary measure used to assess cognitive fusion was the German version of the CFQ (CFQ-D: China et al., 2018). Participants completed the CFQ at T0 and T1 to evaluate changes in cognitive fusion over time.

#### 2.3.4 Statistical analysis

A repeated-measures ANOVA was calculated to examine the effects of group (MBGT + TAU vs. TAU), time (T0 vs. T1), and their interaction on CFQ scores. The analysis focused on within-group (time) and between-group (group) effects, as well as their interaction. Effect sizes are reported as generalized eta squared (ges), which provides a comparable estimate across between- and within-subject designs. As this was an exploratory randomized controlled trial, the sample size was determined by feasibility and is consistent with recommendations for pilot intervention studies, which typically range between 30 and 50 participants (Hayes et al., 2006a,b; Leon et al., 2011). A *post-hoc* power analysis conducted with *G*Power (Version 3.1) indicated th at, given the sample size, a group × time interaction would have required an effect size of approximately *f* = 0.23 ($\eta_{\text{p}}^{2}$ ≈ 0.05) to be detected with a power of 0.80. The significance level was set at α < 0.05 for all statistical tests.

## 3 Results

### 3.1 Study 1: reliability, factor structure, and convergent validity of the CFQ in an international, non-clinical sample of meditators

**Figure 1:**
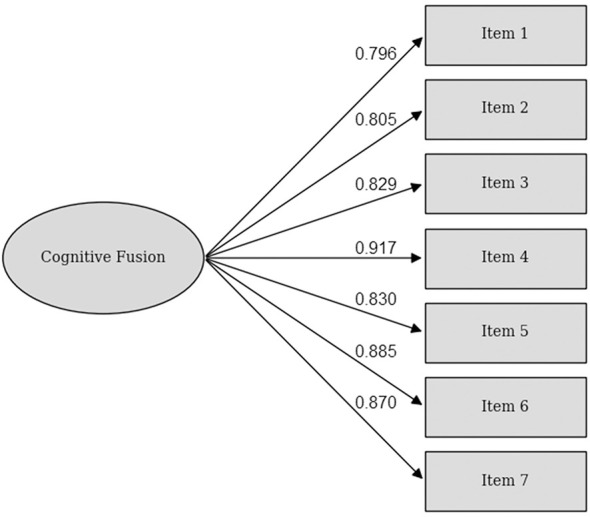
Factor loadings of the CFQ items in an international, non-clinical sample.

A confirmatory factor analysis (CFA) was conducted to evaluate the one-factor structure of the CFQ. The model demonstrated good fit to the data, χ^2^(14) = 104.39, *p* < 0.001, with a Comparative Fit Index (CFI) of 0.98, a Tucker–Lewis Index (TLI) of 0.97, a Root Mean Square Error of Approximation (RMSEA) of 0.09 with a 90% confidence interval (CI) of [0.08, 0.11], and a Standardized Root Mean Square Residual (SRMR) of 0.02. Standardized factor loadings ranged from 0.83 to 0.92, all statistically significant (*p* < 0.001). An overview of the factor loadings can be seen in Figure 1.

Internal consistency was excellent, with α = 0.95 (95% *CI* [0.94, 0.95]). Item-level analyses indicated that removing any single item did not meaningfully improve reliability, with α values ranging from 0.93 to 0.94 across item-deleted models.

Convergent validity was supported by a strong negative correlation between the CFQ and the SMQ, *r* = −0.73, 95% *CI* [−0.76, −0.70], *p* < 0.01, indicating that higher cognitive fusion was associated with lower levels of mindfulness.

### 3.2 Study 2: reliability, factor structure and convergent validity of the CFQ in persons with schizophrenia spectrum disorders

**Figure 2:**
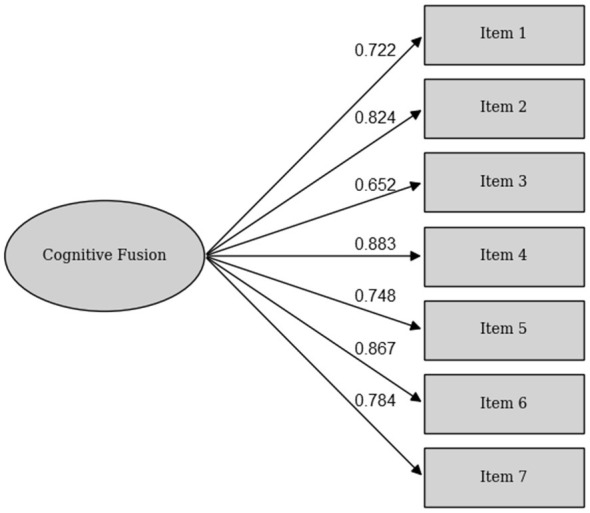
Factor loadings of the CFQ items in persons with schizophrenia spectrum disorders.

A CFA was conducted to evaluate the one-factor structure of the CFQ-D. The model showed acceptable fit, χ^2^ (14) = 34.83, *p* = 0.002, CFI = 0.96, TLI = 0.94, RMSEA = 0.11, 90% *CI* [0.07, 0.16], and SRMR = 0.04. Standardized factor loadings ranged from 0.65 to 0.88, all statistically significant (*p* < 0.001). An overview of the factor loadings can be seen in Figure 2.

Internal consistency was high, with α =0.92 (95% *CI* [0.89, 0.94]). Item-deleted analyses indicated that reliability remained stable across all items, with α values ranging from 0.90 to 0.92.

Convergent validity was supported by a significant negative correlation between the CFQ and the SMQ, *r* = −0.30, 95% *CI* [−0.46, −0.13], *p* < 0.01, indicating that higher cognitive fusion was associated with lower mindfulness in this clinical sample.

### 3.3 Study 3: treatment sensitivity in a randomized controlled trial with patients with schizophrenia spectrum disorders

A total of *N* = 38 inpatients with schizophrenia spectrum disorders were included in the analysis. A repeated-measures ANOVA was conducted to examine the effects of group (MBGT + TAU vs. TAU), time (T0 vs. T1), and their interaction on CFQ-D scores. The main effect of group was not significant, *F*_(1, 36)_ = 0.01, *p* = 0.93, ges < 0.01. There was a significant main effect of time, *F*_(1, 36)_ = 6.70, *p* = 0.01, ges = 0.05. The group × time interaction was not significant, *F*_(1, 36)_ = 1.86, *p* = 0.18, ges = 0.01.

As shown in Table 4, at T0 the MBGT+TAU group had a mean of *M* = 29.10 (*SD* = 10.77), and the TAU group had a mean of *M* = 28.00 (*SD* = 9.37). At T1, the MBGT + TAU group had a mean of *M* = 24.00 (*SD* = 8.77), and the TAU group had a mean of *M* = 26.33 (*SD* = 9.46).

**Table 4:** CFQ mean at baseline and post-intervention and between group differences.

MBGT + TAU		TAU		Between group differences at T1		
T0	T1	T0	T1			
Mean (*SD*)	Mean (*SD*)	Mean (*SD*)	Mean (*SD*)	*F*(d*f*)	*p*	η*p*^2^
29.10 (10.77)	24.00 (8.77)	28.00 (9.37)	26.33 (9.46)	1.50 (1, 35)	0.23	0.04

MBGT, mindfulness-based group therapy; TAU, treatment as usual; T0, baseline; T1, post-intervention; CFQ, cognitive fusion questionnaire; F, *F*-test statistic; df, degrees of freedom; *p*, significance level; η*p*^2^ = partial eta-squared.

## 4 Discussion

The present study examined the reliability, validity, and sensitivity to change of the CFQ in both non-clinical and clinical samples, including participants with SSD. Overall, the findings largely supported our hypotheses regarding the psychometric performance of the CFQ across language versions and populations. As predicted, the scale demonstrated good reliability, a stable one-factor structure, and expected associations with mindfulness in both the non-clinical meditator sample and the clinical SSD samples, supporting its construct validity and transdiagnostic applicability. Consistent with our assumption of population-related differences, psychometric indices were slightly stronger in the non-clinical sample, whereas the SSD sample showed somewhat reduced model fit and weaker associations with mindfulness. Finally, in line with our assumption that cognitive fusion represents a dynamic construct and the CFQ is able to depict changes over time during inpatient treatment, indicating sensitivity to change, although no differential effects between treatment conditions emerged. Together, these findings suggest that the CFQ is a robust measure across contexts while also highlighting clinically relevant considerations when applying it in schizophrenia spectrum disorder samples.

Furthermore, examining specific items within the CFQ that are more challenging for clinical populations may be helpful, as this could offer insights into how cognitive and metacognitive deficits impact item responses. Individuals with SSD typically present with substantial symptom heterogeneity, including fluctuations in cognitive functioning, negative symptoms, and disorganization (Fusar-Poli et al., 2020). Such variability can influence how consistently individuals interpret and respond to self-report items, thereby reducing the coherence of item loadings and attenuating associations with related constructs. Moreover, schizophrenia is characterized by pronounced deficits in metacognition and self-reflective capacity, including difficulties forming integrated representations of one's own thoughts, emotions, and cognitive processes (Lysaker et al., 2007, 2019; Lysaker and Dimaggio, 2014). Because both the CFQ and SMQ rely on introspective access to internal experiences, diminished metacognition may weaken the correspondence between cognitive fusion and mindfulness and contribute to lower factor loadings for items requiring nuanced self-evaluation. Together, these clinical characteristics likely reduce the precision and stability of self-reported cognitive fusion in SSD, explaining the comparatively weaker psychometric performance observed in this sample.

The overall results align with previous research demonstrating the reliability and validity of the CFQ in various populations and confirm the underlying one-factor structure (Gillanders et al., 2014; Viskovich and Pakenham, 2020). Similar studies have found that the CFQ is a robust measure of cognitive fusion, capable of capturing nuanced changes in cognitive processes over time (Gillanders et al., 2014). However, the present findings indicate that the German version of the CFQ may benefit from targeted refinements, particularly to enhance its applicability in clinical populations. Several aspects of the translation warrant closer examination. First, items with comparatively lower factor loadings may require linguistic adjustments to improve clarity and accessibility, especially for individuals with cognitive or metacognitive impairments, which are especially frequent in a subgroup of patients across the psychosis spectrum. This applies most notably to Item 3 (“I over-analyze situations to the point where it's unhelpful”) and, to a lesser extent, Item 1 (“My thoughts cause me distress or emotional pain”). Second, certain German phrasings may not fully capture the experiential nuance intended in the original English items, suggesting that subtle wording modifications could strengthen interpretability across diverse populations. Third, for use in longitudinal clinical trials, it may be beneficial to more explicitly highlight the temporal dimension of cognitive fusion (e.g., “in the past week”), thereby improving sensitivity to change and reducing ambiguity in symptom-fluctuating samples. Together, these considerations may guide future revisions of the CFQ-D to enhance its precision and responsiveness across German-speaking contexts. Our findings extend the existing body of literature by confirming the CFQ's applicability in a clinical sample with SSD, a group that has been underrepresented in prior validation studies (Böge et al., 2021). The moderate fit indices observed in the CFA for the SSD sample, particularly the RMSEA values exceeding 0.10, further underscore the need for careful refinement of selected items. In line with previous studies (China et al., 2018; Gillanders et al., 2014), item 4 (“I struggle with my thoughts”) appears to be an important indicator of cognitive fusion in both the German and English versions, as reflected in its consistently high factor loadings.

The repeated-measures ANOVA revealed a significant main effect of time, indicating that cognitive fusion scores improved across both groups from baseline (T0) to post-intervention (T1). This suggests that cognitive fusion is a dynamic, state-like process that can shift during inpatient treatment. Structured routines, therapeutic contact, medication stabilization, and reduced stressors may all contribute to lower cognitive load, reduced rumination, and improved emotional regulation, which in turn can decrease cognitive fusion. The CFQ-D was sensitive enough to detect this overall improvement, even though the two treatment conditions did not differ significantly. However, neither the main effect for group nor the interaction effect between group and time was significant, suggesting that the reduction in cognitive fusion was comparable for both the MBGT + TAU and TAU groups. The CFQ's sensitivity to change indicates that cognitive fusion is responsive to clinical intervention. While the present studies were not designed to evaluate the effectiveness of mindfulness-based therapies, the observed changes are consistent with the theoretical assumption that such interventions target cognitive fusion as a mechanism of change (Böge et al., 2021, 2022, 2023). Research has shown that sensitivity to change can be influenced by sample size, with smaller samples often lacking the statistical power to detect nuanced effects (Lakens, 2022). Additionally, future research should consider comparing the effectiveness of mindfulness-based therapies and ACT in targeting cognitive fusion. Such comparisons could provide clearer insights into which therapeutic approach is more effective for improving psychological flexibility in clinical populations. These future directions could help refine the CFQ-D and tailor interventions to better address cognitive fusion in schizophrenia spectrum disorder samples. Our findings align with theoretical models that conceptualize cognitive fusion as a core process within psychological flexibility, as reflected in its strong associations with related constructs (Hayes et al., 2012). However, the present data cannot establish centrality in a causal sense, and future research should examine the relative contribution of cognitive fusion within broader process-based frameworks and longitudinal designs which are well powered.

This study's strengths include using a large non-clinical sample and a clinically relevant sample of participants with SSD. However, the study's limitations include its single-center design and the relatively small sample size in Study 3. Future research should examine whether cultural or contextual differences influence the psychometric properties and sensitivity of the CFQ in diverse settings. Considering the study's strengths and limitations, future research should explore the long-term stability of the CFQ and its sensitivity to change over more extended follow-up periods and larger trials (Viskovich and Pakenham, 2018). Additionally, it would be valuable to investigate the CFQ's applicability in other clinical populations and its potential role in predicting treatment outcomes (Hayes et al., 2012). Research into the psychometric properties of specific CFQ items, particularly in diverse populations, could provide insights into potential revisions or adaptations for greater sensitivity.

## Data Availability

The raw data supporting the conclusions of this article will be made available by the authors, without undue reservation.
